# Metallopanstimulin as a marker for head and neck cancer

**DOI:** 10.1186/1477-7819-2-45

**Published:** 2004-12-14

**Authors:** Brendan C Stack, Christopher S Hollenbeak, Christopher M Lee, Frank R Dunphy, Val J Lowe, Paul D Hamilton

**Affiliations:** 1 Division of Otolaryngology-Head and Neck Surgery, Penn State College of Medicine, Hershey, PA, USA; 2 Departments of Surgery and Health Evaluation Sciences, Penn State College of Medicine, Hershey, PA, USA; 3 Department of Health Studies, Lehigh Valley Hospital, Allentown, PA, USA; 4 Department of Radiation Oncology, University of Utah Medical Center, SLC, UT, USA; 5 Department of Medicine, Division of Oncology, Duke University Medical Center, Durham, NC, USA; 6 Department of Radiology, Mayo Clinic, Rochester, MN, USA; 7 James A. Cochran VA Medical Center, St. Louis, MO, USA

## Abstract

**Background:**

Metallopanstimulin (MPS-1) is a ribosomal protein that is found in elevated amounts in the sera of patients with head and neck squamous cell carcinoma (HNSCC). We used a test, denoted MPS-H, which detects MPS-1 and MPS-1-like proteins, to determine the relationship between MPS-H serum levels and clinical status of patients with, or at risk for, HNSCC.

**Patients and methods:**

A total of 125 patients were prospectively enrolled from a university head and neck oncology clinic. Participants included only newly diagnosed HNSCC patients. Two control groups, including 25 non-smokers and 64 smokers, were studied for comparison. A total of 821 serum samples collected over a twenty-four month period were analyzed by the MPS-H radioimmunoassay.

**Results:**

HNSCC, non-smokers, and smokers had average MPS-H values of 41.5 ng/mL, 10.2 ng/mL, and 12.8 ng/mL, respectively (p = 0.0001).

**Conclusion:**

We conclude that MPS-1 and MPS-1-like proteins are elevated in patients with HNSCC, and that MPS-H appears to be a promising marker of presence of disease and response to treatment in HNSCC patients.

## Background

Effective therapy for head and neck squamous cell carcinoma (HNSCC), which constitutes approximately 95% of head and neck malignancies, is dependent upon early diagnosis and intervention. Despite the obvious advantage to earlier diagnosis of head and neck malignancies, no strategy has proven to effectively detect these tumors at early stages. Most head and neck neoplasms are detected when the patient has become symptomatic from the effects of the primary disease or when lymphatic metastases are palpable. These tumors are infrequently found incidentally on physical exam, and in these cases are often discovered at an earlier stage. Stage of disease at time of diagnosis is the primary metric used for determination of therapy and prognostication of life expectancy [1]. As tumor stage advances, the morbidity of surgical resection worsens due to an increased loss of tissue volume and involvement of vital structures. Organ-sparing approaches to head and neck malignancies have been developed in an attempt to treat advanced stage lesions while avoiding conventional surgical morbidities. They have, however, not produced universally superior results to surgery both in terms of local-regional control and function.

Surveillance in the post-treatment head and neck cancer patients has traditionally centered on regular physical examination [2,3]. Office flexible fiberoptic exams of these patients have provided an excellent means of diagnosis for early mucosal recurrences, but are dependent upon the patient's compliance with regular follow-up and often cannot detect submucosal recurrence. Anatomic imaging is used as an adjunct to regular physical exam when recurrence is suspected, when findings are suggestive of cervical lymphatic involvement, or when a patient's symptoms are out of proportion or unexplained by physical exam findings. Imaging of anatomic structures is complicated by alterations in anatomy due to previous surgery or irradiation. Furthermore, despite many promising early reports, no tumor marker has yet been adopted for clinical use which shows high specificity or sensitivity for primary or recurrent HNSCC [4-8].

Metallopanstimulin-1 (MPS-1) was identified, cloned and characterized in the laboratory of Dr. Fernandez-Pol from a cDNA library constructed from a human mammary carcinoma cell line (MDA-468) that was stimulated by the growth factors TGF-β1 and EGF in the presence of cyclohexamide [9]. MPS-1, a multifunctional S27 ribosomal protein, is an 84 amino acid 9.5 kD ribosomal subunit, "zinc finger" protein that is present in all tissues and expressed in large quantities in a wide spectrum of proliferating tissues and oncogenic processes [10-16]. When MPS-1 is over-expressed, it is either secreted or passively released down a concentration gradient into the extra-cellular space.

Conventionally, ribosomal proteins are thought to be confined in their function to intracellular protein synthesis. Many recent reports have drawn attention to "extraribosomal functions" of ribosomal proteins [17-20]. Moreover, these extraribosomal functions have been observed in relation to oncogenesis in various models [21,22]. The zinc finger motif of MPS-1 and other ribosomal proteins may allow binding to nucleic acids which may result in interference with transcription and translation [10,17,18]. Practical applications of this include: 1) DNA repair, 2) gene suppression, 3) cell-cycle control, or 4) control of oncogenesis. Another related ribosomal protein, S27a, is ubiquitinilated and over expressed in human colon cancer. Like MPS-1, it is involved in cell-cycle control and DNA replication [23].

The physiology of MPS-1 expression and our initial experience with this protein in HNSCC has led us to conclude that MPS-1 and MPS-1-like proteins may be useful markers in the effort to screen for and analyze the extent of HNSCC [24]. The purpose of this study was to use an empirical MPS-H test, which measures both MPS-1 and MPS-1-like proteins, to 1) compare average MPS-H levels between HNSCC patients and normal controls and 2) to illustrate how the MPS-H test may be useful for surveillance and evaluating response to treatment for HNSCC.

## Patients and methods

### Patients

A total of 125 volunteers with newly diagnosed HNSCC were prospectively enrolled from a university head and neck oncology clinic. Serum collection consisted of a pre-treatment specimen followed by collections every six weeks during the first year and quarterly during the second year. At the time of specimen collection, presence of HNSCC was judged by all available data including: physical examination (that included an office endoscopy when indicated), biopsy, and radiology [computerized tomography (CT), magnetic resonance imaging (MRI), or positron emission tomography (PET)]. A clinical assessment was rendered based on all available data as "no evidence of disease (NED)", "alive with disease", or an indeterminate status. Serum MPS-H levels in these patients (N = 709) were compared to two control groups. The first control group was comprised of 25 normal, healthy, non-smoking volunteers. The second control group included 64 actively smoking volunteers who were screened for HNSCC and were found to be free of disease during the 1999 Yul Brynner Head and Neck Screening day in St. Louis, Missouri. A total of 821 serum samples collected over a twenty-four month period were analyzed by the MPS-H radioimmuno assay (follow-up days, mean: 217, median: 166.). All patients gave informed consent under an IRB-approved protocol.

### MPS-H Serum Assay

Technical details for the preparation of reagents for MPS-H antigen determinations, RIA procedure, and patient sample preparation are published elsewhere [9,10]. Each serum sample was run in duplicate by a single technician who was blinded to specimen identity. The targets of this assay are the MPS-N-terminus of both MPS-1 and MPS-1-like proteins. These proteins are activated or released from the precursor or carrier proteins by heat-denaturing of the serum under controlled conditions. The resulting proteins are collectively designated as MPS-H. Immunoreactive substances detected by the MPS-H test do not reflect the true levels of authentic immunoreactive MPS-1/S27 ribosomal protein in the circulation under non-denatured conditions.

### Statistical Analysis

Average MPS-H levels were compared between HNSCC patients and the two control groups and across American Joint Committee on Cancer (AJCC) stages and subsites using analysis of variance (ANOVA). Based on the results from the clinical assessment we computed the receiver operating characteristic (ROC) curve for MPS-H. The ROC curve plots the trade-off between sensitivity and specificity for a range of threshold values for defining a positive result. All ANOVA and contingency table analysis were performed using SAS (version 8.1, Cary, NC) statistical software. ROC curve analysis was performed using R software, an open source implementation of the S language (version 1.4.1, ). Statistical significance was defined as p < 0.05.

## Results

Of the 125 HNSCC patients studied, 90 were male (Table 1). Table 2 presents the distribution of stage and site of the primary tumor. Most patients presented in stage III (24.0%) or IV (50.4%) with primary tumors of the oral cavity (26.4%) and larynx (37.6%).

**Table 1:** Demographic and treatment characteristics of cases

**Gender**		**Radiation/Chemo**	**Surgery**	**Total**
Female	*N*	7	28	35
	*%*	5.6%	22.4%	
Male	*N*	19	71	90
	*%*	15.2%	56.8%	
Total	*N*	26	99	125
	*%*	20.8%	79.2%	100.0%

**Table 2:** Summary of primary tumor stage and location.

**Primary**	**Stage**					
**Tumor Site**	**I**	**II**	**III**	**IV**	**Total**	**Percent**
Hypopharynx	0	1	0	1	**2**	*1.6%*
Larynx	7	4	18	18	**47**	*37.6%*
Nasopharynx	0	0	0	4	**4**	*3.2%*
Neck	0	1	1	3	**5**	*4.0%*
Oral Cavity	5	8	4	16	**33**	*26.4%*
Oropharynx	0	0	2	18	**20**	*16.0%*
Parotid	0	0	2	0	**2**	*1.6%*
Skin	4	2	3	3	**12**	*9.6%*
**Total**	**16**	**16**	**30**	**63**	**125**	
**Percent**	*12.8%*	*12.8%*	*24.0%*	*50.4%*	*100.0%*	

**Figure 1:**
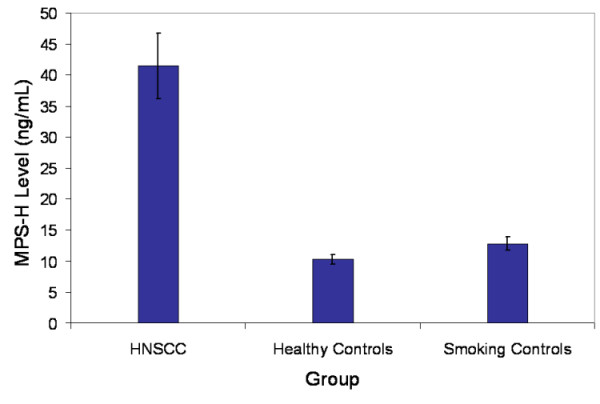
Mean serum MPS-H level for patients with SCC (n = 125, all stages and sites within the head and neck), healthy control group (n = 51) and actively smoking volunteers who were screened for HNSCC (N = 64). Mean of SCC group is 41.5 ng/mL, mean of healthy control group is 10.2 ng/mL, and mean of screening controls is 12.8 ng/mL (p < 0.0001). Error bars denote 1 standard deviation.

**Figure 2:**
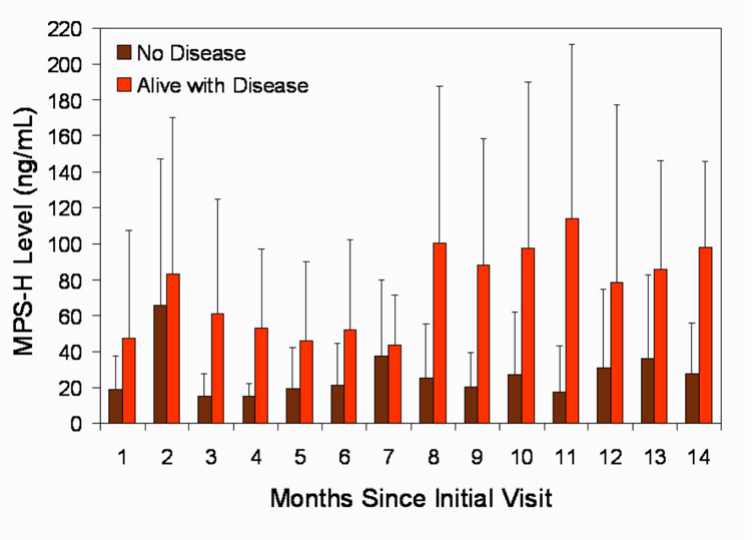
Serial MPS-H levels in HNSCC patients treated and without clinical disease (No Disease) and patients with unresectable disease or receiving palliative therapy with persistent clinical disease (Alive with Disease). Error bars denote 1 standard deviation and vary widely in the AWD group due to its small size and patient attrition over time from death.

MPS-H levels in this group were compared to a control group of healthy volunteers and to a control group who volunteered for screening for head and neck cancer (Figure 1). Mean MPS-H was 10.2 ng/mL for the healthy control group, and 12.8 ng/mL for the smoking control group. Mean MPS-H for the HNSCC group was 41.5 ng/mL, which was significantly higher than both control groups (p < 0.0001). Furthermore, Figure 2 illustrates that among HNSCC patients, those who were successfully treated and clinically free of disease had consistently lower MPS-H levels over time than patients living with active head and neck cancer.

**Figure 3:**
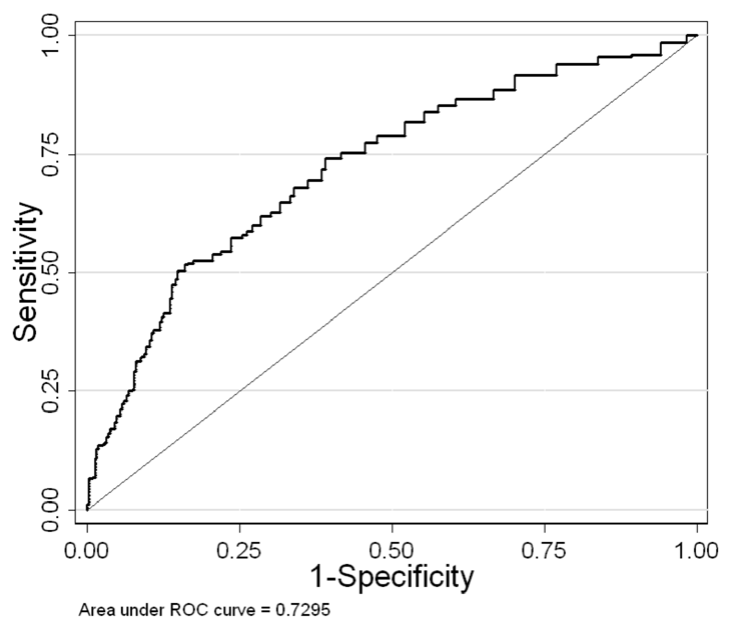
Receiver operating characteristic (ROC) curve for MPS-H test. Area under the curve is 0.73 (CI_95_: 0.69 – 0.76, p = 0.001)

We next computed the receiver operating characteristic (ROC) curve for the MPS-H levels. Figure 3 shows that the area under the ROC curve (0.73; 95% CI: 0.71–0.75; p = 0.001), is significantly different from 0.5, which suggests that there is moderate diagnostic accuracy associated with MPS-H. Analysis of MPS-H levels as a function of AJCC stage or head and neck sub site were performed and were not significant. Larger numbers of earlier stage (I and II) tumors and greater numbers among the various sub sites might result in significance.

**Figure 4:**
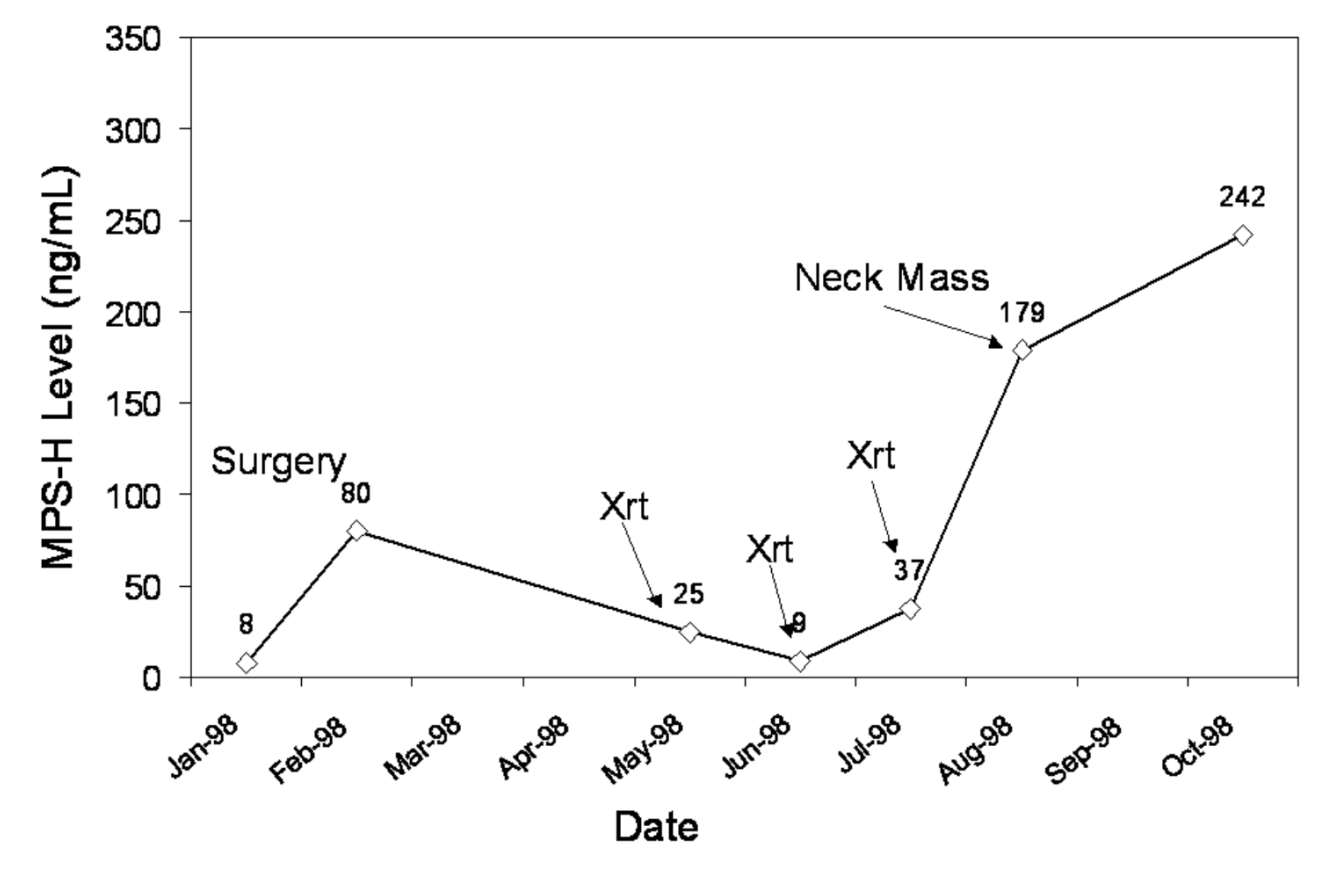
Patient without evidence of disease from surgery through radiation therapy (draws 1–5). The patient suffered a recurrence of clinical disease following radiation therapy (draws 6–8), which progressed until death.

**Figure 5:**
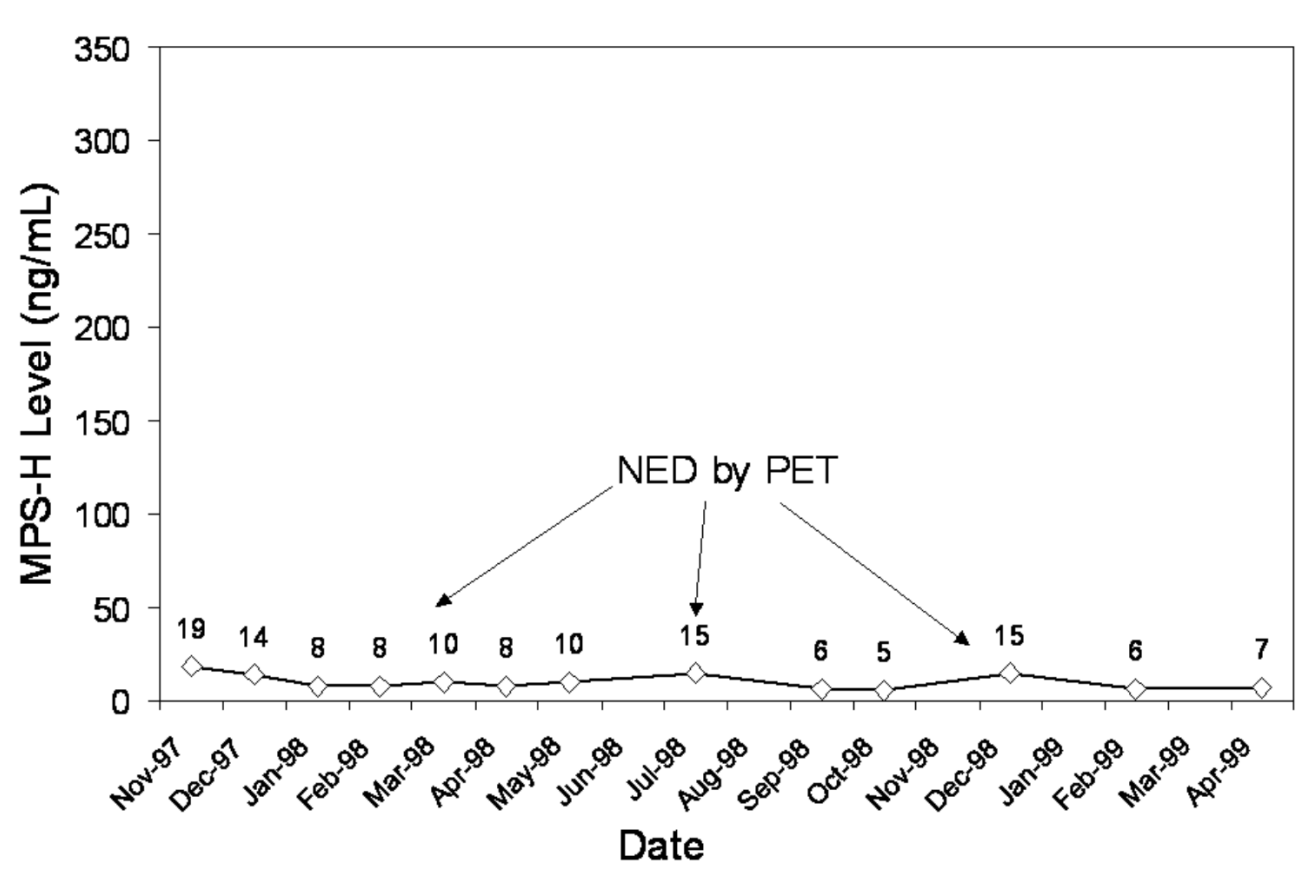
Patient followed for 24 months without evidence of recurrence on physical exam, endoscopy, or FDG-PET.

**Figure 6:**
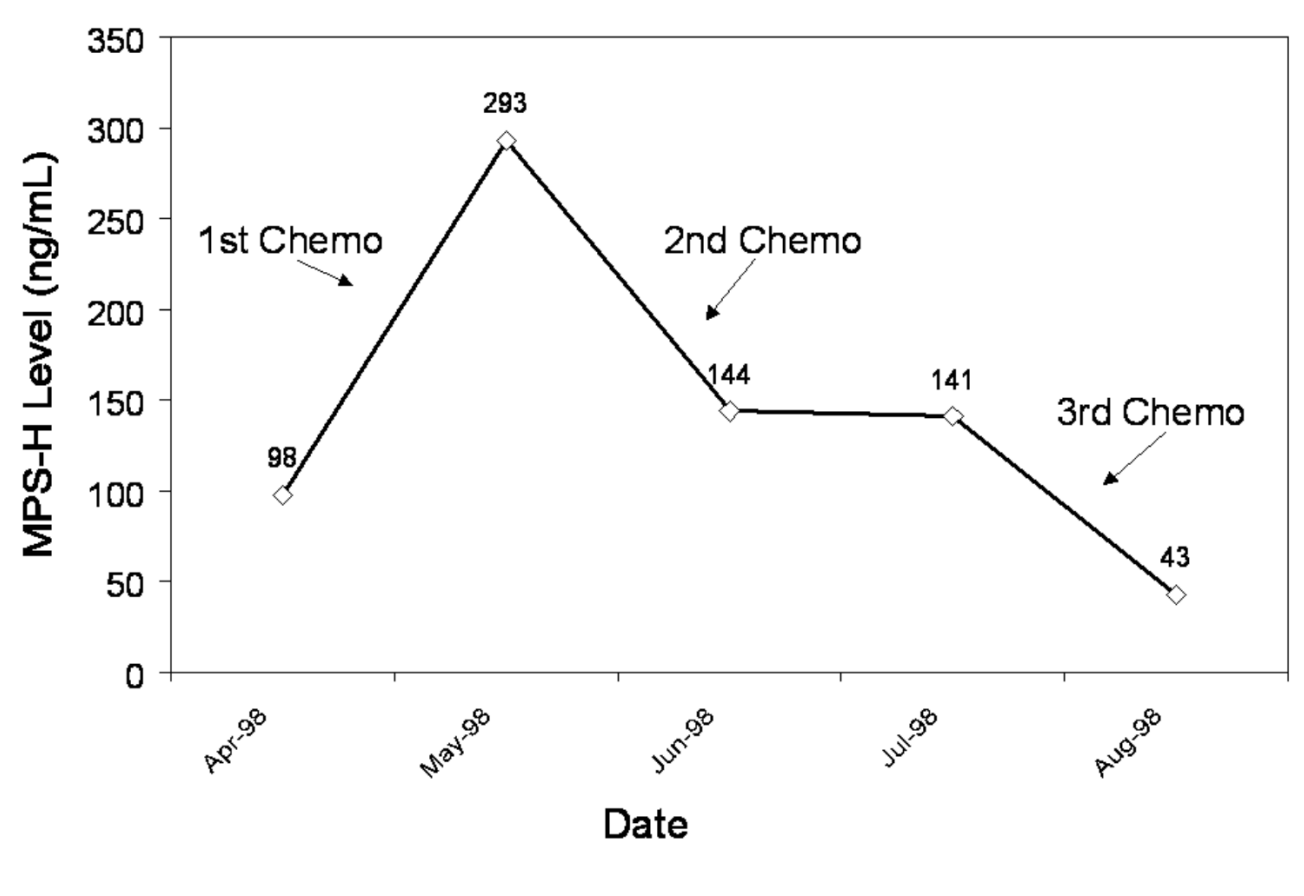
Patient with unresectable tumor. Note MPS-H level response to chemotherapy. Patient subsequently expired after refusing further therapy.

We have observed several instances where elevated MPS-H levels in patients presenting with head and neck neoplasms dropped to normal levels following successful therapy. We have also noted examples of persistent elevations or increases in MPS-H levels in patients with failure to respond to therapy or with recurrence of tumor respectively. Several cases of patients presented in Figures 4, 5, 6 illustrate these phenomenons. Patient 1 (Figure 4) was a female with a T_4_N_0_M_0 _SCC of the floor of mouth with positive tumor margins on the cut edge of the mandible, having failed a recent limited surgery by another surgeon. Her presenting MPS-H value was borderline positive when she had little clinical disease [1 on the x axis] and rose immediately postoperatively. A transient rise after surgical ablation or induction chemotherapy is a documented phenomenon observed with numerous tumor markers (personal communication J.A. Fernandez-Pol). The elevation likely results from a large initial disruption of cells within the tumor resulting in a dumping of intracellular MPS-1 into the circulation. This usually returns to baseline in 4 to 6 weeks if the tumor is successfully treated. The patient was clinically NED in the immediate postoperative period and during radiation treatment. Approximately 6 months post operatively, the patient developed a neck mass and by the next visit skin metastasis were seen. On last follow-up, the patient had progression of skin and neck metastasis at which time she decided to pursue hospice care. She expired 6 weeks later.

Patient 2 (Figure 5) is a male who was diagnosed with a T_4_N_2c_M_0 _SCC of the larynx, which required total laryngectomy, bilateral neck dissections, and postoperative radiation therapy. The patient joined this study 3 months following his surgery, while clinically NED, and has remained free of clinical recurrence for 24 months. The patient had three FDG-PET scans at six-month intervals following his surgery that were all negative for recurrent disease or metastasis. His persistently low MPS-H levels over time (range from 7.7–19.2 ng/mL), was suggestive of ongoing disease-free status.

Patient 3 (Figure 6) was a female who presented with a T_4_N_0_M_0 _SCC of the hypopharynx. She was severely malnourished with multiple medical problems and thought to be a poor surgical candidate. She decided to pursue induction chemotherapy (Carboplatin and Taxol) to be followed by radiation therapy. She underwent three rounds of chemotherapy at 21-day intervals and had a 50% decrease in her tumor size as judged by office endoscopy. She suffered severe GI problems during her therapy, opted not to continue on to radiation, and enrolled in hospice care. The patient did not present for additional follow-up after entering hospice and died three months later. This case illustrates the potential utility of MPS-H as a marker for tumor response to chemotherapy and/or irradiation.

## Discussion

Histological examination of the tumor, surgical margins, and cervical nodes are the current means of determining extent of disease. When surgical extirpation is not undertaken, staging is performed based on a radiological assessment, biopsy, and physical examination. These methods are used either to determine adequacy of resection and the need for adjuvant therapy or to select an alternative primary (non-surgical) therapy, respectively. Limitations of the histologic method include microscopic disease that escapes diagnosis due to a small number of malignant cells, subtle histological changes not classified as cancer, previous treatment effect upon tissues, or pathologic sampling error. CT and physical examination both suffer from modest sensitivity and specificity in detecting many early head and neck neoplasms. As a result, local and regional treatment failures are not uncommon in both surgical and non-surgical treatments of head and neck cancer. This may be due to an underestimation of the tumor burden. This may also be explained by the current diagnostic emphasis upon analysis of structure (microscopic) or anatomic extent of disease, both of which are imperfect, rather than its biologic activity as might be measured by a tumor marker or a functional scanning technique such as FDG-PET.

The MPS-H test has been used in conjunction with conventional tumor-specific markers to improve sensitivity and specificity of tumor serodiagnosis [12]. Of all malignancies in which MPS-H has been studied to date, epithelial malignancies possess no alternative tumor markers in clinical use that have been effective for diagnosis or surveillance [4-8,23]. This observation in conjunction with the current dependence on anatomic evaluation for diagnosis of epithelial malignancies has led us to preliminary investigations of the utility of MPS-H serologic diagnosis for the detection of head and neck epithelial neoplasms.

Control groups of healthy volunteers and those with systemic, non-malignant diseases have been studied using the MPS-H test. Statistical analysis has revealed that those without malignancy have MPS-H serum levels less than 10 ng/mL, those with malignancy have levels greater than 20 ng/mL, and those with bony metastasis have levels in excess of 100 ng/mL [12]. Additionally, MPS-H levels have been documented to decline with successful treatment of malignant disease whereas non-responders to therapy persisted with high levels of MPS-H [12].

Serum samples from prostate carcinoma patients with high levels of MPS-H (>500 ng/mL) have demonstrated the authentic 9.4 kDa MPS-1 protein, at least one protein with sequence homology to the N-terminus of MPS-1, and a high molecular weight precipitation interfering protein.

More accurate detection of recurrent SCC of the head and neck by the MPS-H test may provide a new way to improve survival. Physical exam, conventional imaging, and biopsy are the current gold standard to determine recurrence. Currently, FDG-PET is the most promising way to assess early tumor recurrence of the head and neck but is quite expensive [25,26]. Its sensitivity and specificity have been reported to be approximately 90% [25,26]. No standard serum tumor marker is routinely used for head and neck cancer surveillance, which limits alternatives to conventional exams or frequent FDG-PET imaging [4-8,25].

In the present study we compared FGD-PET to MPS-H levels in head and neck cancer patients. FDG-PET interpretation and MPS-H level determination were performed independently and blinded from the results of the other test. FDG-PET positive scans were not all confirmed by biopsy in our study. A statistically significant correlation was noted between FDG-PET positive cases and high MPS-H serum levels in head and neck cancer patients. MPS-H and FDG-PET agreed in 103 out of 183 cases. In 12 cases MPS-H was elevated but no cancer was found by FDG-PET, suggesting that the patients may have had an early recurrence detectable by MPS-H but not yet by FDG-PET. The 68 FDG-PET positive cases that show low MPS-H levels suggest that some tumors were unable to produce high levels of MPS-H, perhaps due to previous chemotherapy, or that some results represent false positive PET scans. Further study with larger patient groups is ongoing to assess the optimal cutoff levels of MPS-H and the correlation between FDG-PET and MPS-H.

Shortcomings of this study include a limited time span, a large proportion of advanced stage cancers, limited controls (both size, smoking status, and age/gender match), and ability to define an absolute cut off value for normal vs. abnormal. We recognize that other factors such as age and other malignancies may effect MPS levels. Future studies will attempt to address and/or control for these issues.

Considering the data presented in this paper, which agrees with previous results with other tumor types, and the compelling need to expedite the early diagnosis of primary and recurrent epithelial malignancies of the head and neck, we are further evaluating the MPS-H tests as a tool for diagnosis in a larger group of HNSCC patients. Additionally, we are working to improve the diagnostic technology used to detect MPS-H [27-29]. Since there is a reasonable correlation between detection of MPS-H in the sera and FDG-PET positivity for SSC, these results raises the potential of the MPS-H test for becoming a test for HNSCC, followed by selective confirmatory FDG-PET imaging. These preliminary results will be verified in a larger population. The role for using MPS-H as a general screening tool among at-risk populations without the diagnosis of HNSCC is also currently being evaluated.

## Competing interests

The authors declare that they have no competing interests.

## Authors' contributions

BCS: Principal Investigator, Principal Editor

CSH: Data analysis and manuscript preparation

CL: Medical student research assistant for project

FD: Member of multidisciplinary head and neck oncology team, recruiter of subjects

VJL: Member of multidisciplinary head and neck oncology team, supplier of PET data, data analysis.

PDH: Laboratory technical support with MPS assay

## Funding sources

This study was funded by the clinical salaries of BCS, FRD, and VJL. CML and CSH were students at the time of this study. MPS assays were performed at the Department of Veterans Affairs James A. Cochran Medical Center, Molecular Oncology Laboratory, St. Louis, MO. PDH was an employee of the US Government at the time of this research.
